# Renal cell carcinoma and upper tract urothelial carcinoma in kidney transplant recipients

**DOI:** 10.25122/jml-2025-0065

**Published:** 2025-04

**Authors:** Oana Moldoveanu, Cătălin Baston, Bogdan Sorohan, Lucas Discalicău, Ioanel Sinescu

**Affiliations:** 1 Department of Urology, Carol Davila University of Medicine and Pharmacy, Bucharest, Romania; 2 Fundeni Clinical Institute, Center of Surgical Urology and Kidney Transplantation, Bucharest, Romania; 3 Department of Nephrology, Carol Davila University of Medicine and Pharmacy, Bucharest, Romania

**Keywords:** renal cell carcinoma, urothelial carcinoma, upper tract urothelial carcinoma, kidney transplant, immunosuppression, end-stage kidney disease, acquired cystic disease

## Abstract

Renal cell carcinoma (RCC) is the most common solid-organ malignancy in Western countries, and upper tract urothelial carcinoma (UTUC) is the most common malignancy in Asian countries. The management of RCC/UTUC in kidney transplant recipients is complex and clinically challenging due to post-transplant modifications associated with immunosuppressive treatment. This retrospective study evaluated the incidence, risk factors, treatment outcomes, and oncological implications of RCC and UTUC in kidney transplant recipients from 2008 to 2023. Data were collected from clinical records, and follow-up calls for 20 patients diagnosed with RCC and UTUC among 2,283 kidney transplant recipients, revealing an incidence rate of 0.78% for RCC (18 patients) and 0.087% (two patients) for UTUC. Most patients presented localized disease at diagnosis. Surgical interventions included radical nephrectomy for the native kidney’s RCC, radical or partial nephrectomy for allograft RCC, and radical nephroureterectomy for UTUC in the native kidney and allograft. Oncological outcomes indicated a mean follow-up of 51.29 months, during which five patients (25%) developed metastases, which achieved prolonged survival through surgical management, adjuvant therapy, and immunosuppression adjustments. The study highlights the increased cancer risk in this population and underscores the necessity for established screening protocols and individualized treatment strategies to optimize patient outcomes while preserving kidney function. These findings contribute to the ongoing research on managing malignancies in transplant recipients, with implications for further research and clinical guidelines.

## INTRODUCTION

Kidney transplant (KT) is the treatment of choice for patients with end-stage kidney disease (ESKD), significantly improving overall survival (OS). KT offers the advantage of a lower complication rate than patients who remain on dialysis [1-3]. Advances in surgical techniques, postoperative care, and the development of new immunosuppressive agents have contributed to longer graft survival and significant improvements in quality of life. However, the risk of developing malignancies remains high in this population [1,4-7]. Renal cell carcinoma (RCC) is the most prevalent solid-organ malignancy, with a 5- to 10-fold higher risk than in the general population, and represents 6.8% of all malignancies in this population [7-9]. In 90% of the cases, RCC is located in the native kidneys, rarely in the kidney graft [7]. Urothelial carcinoma (UC) is typically localized in the bladder, but upper tract urothelial carcinoma (UTUC) of the native kidneys or kidney grafts is less common in Western countries compared to Asia. In Asia, UTUC in native kidneys is the most frequent malignancy following KT [10-16].

Radical nephrectomy or nephroureterectomy remains the treatment of choice for RCC and UTUC in non-functional native kidneys [16-20]. Diagnosing and treating RCC and UTUC in kidney grafts is particularly challenging, as it requires balancing the preservation of renal function with optimal oncologic outcomes. These malignancies in kidney transplant recipients are further complicated by local anatomical changes, transplant-related risk factors, and underlying ESKD-related factors [16-25]. Managing these types of cancers in kidney transplant recipients is a complex process that demands an experienced, multidisciplinary approach to ensure personalized treatment. To date, there are no established guidelines or clear recommendations for the therapeutic management of these patients. This study aimed to examine the incidence, risk factors, surgical outcomes, and survival rates for kidney transplant recipients with RCC and UTUC.

## MATERIAL AND METHODS

### Study design and participants

This retrospective study evaluated kidney transplant recipients diagnosed with RCC or UTUC in either the native kidneys or the kidney graft between 2008 and 2023. Patient data was collected using the Fundeni Clinical Institute Hipocrate platform, patient record files, and follow-up calls. Inclusion criteria were kidney transplant recipients diagnosed with renal cell carcinoma and upper tract urothelial carcinoma who underwent radical or partial nephrectomy or nephroureterectomy of native kidneys or kidney graft. The functional status of the graft was defined according to the need to initiate dialysis in functional and non-functional grafts. Exclusion criteria were patients diagnosed with post-transplant lymphoproliferative disorder (PTLD) affecting kidneys, patients who did not receive active treatment for RCC or UTUC, patients who received partial or radical nephrectomy or nephroureterectomy for other causes, and patients diagnosed with other types of malignancies pretransplant.

### Patient data

Clinical and demographic data collected for this study included age, sex, cause of ESKD, duration of dialysis before transplantation, and presence of acquired cystic kidney disease (ACKD). Additional variables included history of previous kidney transplantation, non-specific pre-transplant risk factors (such as smoking status, hypertension, analgesic exposure, obesity, history of infections, and history of urinary lithiasis), year of transplantation, and donor type. Information regarding the immunosuppressive regimen prior to malignancy diagnosis, graft function status, post-transplant infections, and occurrence of other post-transplant malignancies was also recorded. Data related to the timing and type of surgery for malignancy (radical nephrectomy, partial nephrectomy, or nephroureterectomy), postoperative complications, tumor characteristics (histopathology, staging, and grading), changes in immunosuppression following malignancy, follow-up period, time to metastasis, treatment of metastasis, and time and cause of death were systematically collected for analysis.

### Outcomes

The primary endpoint of the study was overall survival, defined as the time from the date of surgery for RCC or UTUC to the date of death or the last follow-up before June 1, 2024. Overall survival at over 10 years of follow-up was evaluated using Kaplan–Meier survival analysis.

## RESULTS

Between 2008 and 2023, among 2,283 patients who underwent kidney transplantation, twenty-one recipients were diagnosed with RCC or UTUC. One patient was excluded due to refusal of nephrectomy for a cT3aN1M1 RCC (low-volume pulmonary metastasis) of the left native kidney with functional kidney graft. Twenty patients were included in the study. Among them, three patients (15%) were diagnosed with kidney graft tumors (one patient with RCC on functional graft, one patient with RCC in low functional graft, and one patient with allograft UTUC), and 17 (85%) patients with native kidney tumors (one patient with bilateral RCC, one patient with unilateral UTUC).

Most patients diagnosed with RCC post-KT were men (14 out of 20, 70%). The mean age of kidney transplant recipients was 45.55 years (range 28–61). Most kidney grafts were obtained from deceased donors (13/20, 65%). One patient (5%) developed RCC following a second transplant from a living donor. None of the deceased or living donors had a history of malignancy, and no living donor subsequently developed urologic or other types of malignancies.

The mean age of developing renal tumor after KT was 52.65 years (51.22 years for RCC and 65.5 years for UTUC). The evaluated risk factors are presented in Table 1. The mean dialysis time before KT was 5.1 years (range 0 to 14 years). Eight patients presented ACKD prior to KT and developed RCC in native kidneys (8/16, 50%). Immunosuppression regimens included calcineurin inhibitors for all patients. The mean time of immunosuppression was 7.07 years (range 3 months to 18 years). Six patients were diagnosed with RCC in native kidneys with non-functional grafts at the time of diagnosis (6/16, 37.5%), and one patient was diagnosed with RCC in a non-functional transplanted kidney (1/3, 33.33%). Both patients with UTUC had functional grafts at the time of malignancy detection.

**Table 1:** Patient’s characteristics

	Sex	Age KT	Cause of ESKD	Donor type	Dialysis pre-KT (y)	Risk factors	Age	Diagnostic	Renal tumor location	Type of renal tumor	Graft function	Immunosuppression time before renal tumor (years)
Case 1	F	34	N	D	3	HTA Obesity	54	Hematuria Abd. pain	G	ccRCC	F	18
Case 2	M	41	ADPKD	L	2	Smoking Obesity	45	Inflammatory	G	cc-pRCC	Non-F	5
Case 3	M	52	Obstructive uropathy	D	10	HTA Analgesic consumption	64	Hematuria Abd. Pain Inflammatory	G	UTUC	F	10
Case 4	M	48	Glomerulopathy	D	3	HTA Obesity ACKD BKV	50	I	NK	ccRCC	Non-F	1
Case 5	F	53	N	D	2	HTA Obesity	60	I	NK	pRCC	F	8
Case 6	M	28 (1^st^) 36 (2^nd^)	Glomerulopathy	L	0	HTA	42	I	NK	ccRCC	Non-F	15
Case 7	M	35	Glomerulopathy	L	14	HTA Smoking ACKD	42	I	NK	ccRCC	Non-F	3
Case 8	F	28	Uropathy	L	13	HTA ACKD	51	I	NK	ccRCC	Non-F	10
Case 9	F	61	Uropathy	D	8	HTA ACKD	63	Hematuria Abd. pain	NK	ccRCC	F	3
Case 10	M	30	Glomerulopathy	D	0	HTA	36	Incidental	NK	pRCC multiple	F	11
Case 11	M	48	Glomerulopathy	L	4	HTA Smoking ACKD	49	I	Bilat. NK	ccRCC	F	0.4
Case 12	M	37	N	L	2	HTA Obesity	50	I	NK	ccRCC	F	14
Case 13	F	54	Glomerulopathy	L	12	HTA Obesity	64	Hematuria	NK	ccRCC	F	10
Case 14	M	50	ADPKD	D	0	No	59	I	NK	pRCC	F	9
Case 15	M	55	N	L	2	HTA	67	Abd. Pain Inflammation Hydronephrosis	NK	UTUC	F	12
Case 16	M	44	N	D	5	HTA	46	I	NK	ccRCC	F	2
Case 17	M	45	N	D	5	HTA	47	I	NK	ccRCC	F	2
Case 18	F	57	Glomerulopathy	D	6	HTA ACKD Obesity	58	I	NK	ccRCC	F	2
Case 19	M	31	Glomerulopathy	D	6	HTA ACKD Obesity	47	I	NK	chRCC	Non-F	5
Case 20	M	60	Glomerulopathy	D	5	HTA ACKD	61	I	NK	ccRCC	Non-F	1

KT, Kidney Transplant; N, Undetermined; ADPKD, Autosomal Polycystic Kidney Disease; HTA, Hypertension; D, Deceased Donor; L, Living Donor; I, Incidental; NK, Native Kidney; G, Graft; F, Functional Graft; Non-F, Non-Functional.

### Surgical aspects

A retroperitoneal open radical nephrectomy was performed for most cases of RCC in native kidneys (15/20 patients, 75%). In one case of bilateral RCC, a laparoscopic simultaneous bilateral nephrectomy was performed (5%). For RCC involving the kidney allograft, retroperitoneal transplantectomy was performed in one patient (Case 2) and open partial nephrectomy in another (Case 1). In the case of UTUC involving the kidney graft (Case 3), radical nephroureterectomy with bladder cuff excision was performed. Similarly, for native upper tract UC (Case 15), radical nephroureterectomy was carried out. Only five patients (25%) presented Clavien–Dindo grade II complications, and no patient developed grade III or higher complications in the first month after surgery.

### Pathology findings

Clear cell RCC (ccRCC) was the most common histological subtype, identified in both native kidneys and, in one case, within the allograft (13/20, 65%). Papillary RCC was found in native kidneys in four patients (20%), while a mixed papillary-clear cell subtype was detected in the kidney graft in one patient (Case 2, 5%). In Case 19, an initial suspicion of oncocytoma was revised after a second pathology review, confirming a diagnosis of chromophobe RCC (chRCC, 5%). Regarding tumor grading, Fuhrman grade 3 was observed in five patients (25%), while most RCC cases were low-grade (Fuhrman grade 1–2, 65%). Both UTUC cases (10%) were identified as high-grade, muscle-invasive tumors: one was staged as pT4 in the transplanted kidney and the other as pT2 involving the renal pelvis and distal ureter of the native upper urinary tract.

### Oncological outcomes

The oncological outcomes are summarized in Table 2. The mean follow-up for all patients was 56.9 months, ranging from 6 months to 140 months, and consisted of regular ultrasound examinations, CT scans, and evaluations of kidney function.

Two patients (10%) who had RCC in native kidneys developed metastases during follow-up, with a mean time to metastasis of 6.5 years after radical nephrectomy. One patient (5%) developed local recurrence at 4 years post-surgery, another patient (5%) was diagnosed with RCC in the contralateral kidney at 5 years, and one patient (5%) had pulmonary metastases detected at the time of nephrectomy.

**Table 2:** Oncological outcomes after surgical treatment of renal tumors

Parameter	Value
Type of surgery	G-RN 1/20 (5%) G-NSS 1/20 (5%) G-NFU 1/20 (5%) RN 15/20 (75%) NFU 1/20 (5%) LARN bilateral 1/20 (5%)
Clavien-Dindo complications at the time of surgery	I (14/20, 70%), II (6/20, 30%)
RCC stages and grading	pT1a G 2/20(10%) pT1a NK 5/20 (25%) pT1b NK 4/20 (20%) pT2a NK 1/20 (5%) pT3a NK 5/20(25%) Unclassified NK 1/20 (5%) Multilocular NK and G (2/20, 10%) Fuhrman 2 NK 11/20 (55%) G 2/20 (10%) Fuhrman 3 NK 5/20 (25%)
UTUC stages and grading	pT2 LG – native kidney (1/20, 5%) pT4 HG – kidney graft (1/20, 5%)
Follow-up	57.2 months (range 6 to 140 months)
Metastasis sites (6 metastatic patients)	Extra-regional LN 1/20 (5%) Pulmonary 2/20 (10%) Prostatic 1/20 (5%) Local recurrence 1/20 (5%) Metachronous RCC in contralateral kidney 1/20 (5%)
Cause of death (4 patients died)	Cancer-related death 1/20 (5%) COVID-19 3/20 (5%)

G-NSS, Graft Nephron-Sparing Surgery; G-RN, Graft Radical Nephrectomy; RN, Radical Nephrectomy of Native Kidneys; G-NFU, Graft Nephroureterectomy; LARN, Laparoscopic-Assisted Radical Nephrectomy; NK, Native Kidney; G, Graft; LN, Lymph Node.

Among the patients who developed metastases, one (5%) developed ccRCC metastasis localized to the prostate and seminal vesicle at 7 years post-nephrectomy, confirmed by biopsy. The patient received tyrosine-kinase inhibitors (TKIs) and maintained stable disease for 4 years with a functioning kidney graft. During the immunosuppression regimen, tacrolimus was changed to sirolimus after metastasis detection. Unfortunately, the patient died due to COVID-19 infection after 4 years. Another patient (5%) developed extra-regional lymph node metastases 6 years after surgery. No adjuvant treatment was administered, and sirolimus was added to the immunosuppression regimen. This patient also died of COVID-19 infection 5 years later, with progressive metastatic disease but a functional allograft. The patient with chRCC developed local recurrence four years after radical nephrectomy of the native kidney, requiring surgical resection. A second local recurrence occurred after another four years without distant metastasis. Surgery for the retroperitoneal tumor (stage IV with liver invasion) was performed; however, the patient died 5 days postoperatively due to cancer-related complications. The pathology findings after both tumor recurrences confirmed the chRCC histology. At the time of the native kidney nephrectomy, the patient was on dialysis after an allograft nephrectomy for a non-functional graft one year earlier. One patient developed contralateral RCC five years after undergoing native kidney nephrectomy for clear cell RCC (pT1aN0M0). This patient had returned to dialysis one year after transplantation due to chronic graft dysfunction. The patient refused contralateral nephrectomy and remained alive at the time of the study.

One case of urothelial carcinoma of the transplanted kidney was detected 10 years after KT. Initially, the patient was diagnosed with bladder UC and treated with TURBT for multiple pTa low-grade tumors. Bacillus Calmette–Guérin (BCG) therapy was initiated and maintained for one year without adverse reactions or recurrence. No changes to immunosuppression were made during BCG instillation. Two years later, hematuria and iliac fossa pain prompted reevaluation. The initial suspicion of BCG pyelonephritis was not sustained by the urinary tests. A kidney graft biopsy was performed, confirming the UC of the allograft. The CT scan detected pulmonary metastasis. A nephroureterectomy of the allograft with bladder cuff was performed, and the pathology exam showed pT4HG (pelvicalyceal urothelial carcinoma with sinus and perinephric fat invasion and psoas muscle involvement). The patient returned to dialysis, withdrawal of immunosuppression, and spontaneous remission of pulmonary metastasis was confirmed 3 months after surgery, with no adjuvant therapy. The patient is still alive, with neither bladder recurrence nor distant metastasis at 36 months follow-up.

Regarding immunosuppressive management, 10 patients (50%) underwent no changes. Conversion from tacrolimus to sirolimus occurred in three cases (15%), while in one case (5%), cyclosporine was reduced, and sirolimus was added. In two cases (10%), tacrolimus dose reduction was performed. In four cases (20%), immunosuppression was completely withdrawn. None of these adjustments negatively impacted graft function.

**Figure 1:**
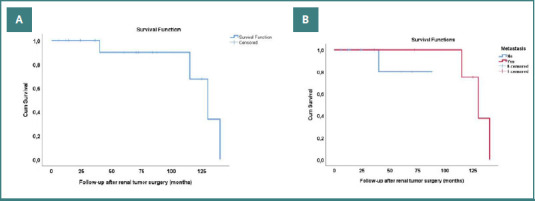
Kaplan-Meier analysis for patients’ survival. A, Overall survival; B, Survival according to the presence/absence of metastasis.

At study completion, 15 patients (75%) were alive, with 14 of them (70%) free from tumor recurrence or distant metastases (Figure 1). Three patients (15%) were on dialysis at the time of nephrectomy for RCC, one patient (5%) returned to dialysis after transplantectomy for RCC, and another patient (5%) after nephroureterectomy for UTUC of the allograft. No patient underwent re-transplantation following surgical treatment of malignancy.

## DISCUSSION

The incidence of RCC in kidney transplant recipients is significantly higher than in the general population (0.7% vs. 0.04%). RCC affecting the native kidneys is the most common presentation [7,25-29]. In our study, 80% (16/20) of cases involved RCC in native kidneys, while RCC in the kidney graft was identified in two cases (10%). The most important risk factors associated with RCC in kidney transplant recipients are male sex, male donor sex, history of ACKD, duration of dialysis prior to kidney transplant, history of RCC, smoking, obesity, hypertension, immunosuppression and donor-derived malignancies [4,5,30]. Sapir *et al*. found an elevated risk of primary malignant neoplasms in female patients receiving higher cumulative doses of mycophenolate, as well as in young male patients exposed to tacrolimus over an extended period [5].

The cumulative incidence of primary malignancies rises with time post-transplantation, reaching 4–5% at 5 years, 10% at 10 years, and 25% at 20 years [4]. ESKD, as well as its etiology, significantly contributes to this risk, with a 3.6-fold increased likelihood of developing RCC compared to the general population. ESKD due to vascular or glomerular disease, or hypertensive nephrosclerosis, is associated with a higher risk of RCC than ESKD resulting from diabetes or polycystic kidney disease [1,31,32]. Approximately 60% of patients undergoing dialysis for 2–4 years develop ACKD, and among these, 20% may eventually develop RCC. The documented involution of the cysts after kidney transplant may suggest a protective role of transplant for developing RCC in patients with ACKD [33-35]. However, the risk of RCC in the kidney transplant population is higher than in the general population, suggesting that not just ACKD is related to RCC pathogenesis and that the post-transplant risk factors may play an important role in the process [33-35].

In our study, we observed that ccRCC was the predominant subtype RCC (70%) encountered in both native kidneys and, in one case, within an allograft, followed by pRCC (20%), occurring mostly in male patients. These findings agree with other studies [23,36,37]. All RCCs in our study were Fuhrman grades 2–3. Localized tumors (stages T1–T2) were more common (65%); however, 25% (5/20) of RCCs arising in native kidneys were stage T3a. This finding differs from data previously published [5,23,24,37].

The incidence of UC is also higher in kidney transplant recipients, with a threefold increase compared to the general population [38]. Risk factors for UC include exposure to aristolochic acid (from Chinese herbs or Balkan endemic nephropathy), BK virus (BKV) reactivation, smoking, and occupational exposures [39-41]. Data regarding UTUC in native or graft kidneys remain limited to small studies and case reports. In our study, both cases of UTUC were high-grade, muscle-invasive tumors (10%), consistent with findings by Zhang *et al*. in a Chinese cohort [16].

Currently, there are no guidelines for managing or screening RCC or UC in kidney transplant patients. The 2020 KDIGO Clinical Practice Guidelines on the Evaluation and Management of Candidates of Kidney Transplantation recommend screening candidates at increased risk for RCC (e.g., dialysis for more than three years, ACKD, or analgesic nephropathy) via ultrasound [42]. In our study, 55% (11/20) of renal tumors were diagnosed incidentally during routine post-transplant follow-up, supporting the potential value of structured screening programs.

Radical nephrectomy is recommended for RCC in native non-functional kidneys, for RCC in kidney grafts, for locally advanced or metastatic RCC, for multicentric papillary RCC, or in cases of non-functional grafts in order to ensure good oncologic outcomes [43-48]. Nephron-sparing surgery is preferred for T1a tumors (<4 cm) in functional grafts, achieving favorable oncological outcomes while preserving graft function [21,22,24,49,50]. Open partial nephrectomy for a T1a tumor of kidney graft offered a good oncologic outcome and a functional kidney graft at 36 months follow-up in Case 1. For UTUC occurring in native non-functional kidneys, radical nephroureterectomy with bladder cuff is preferred given the typically high-grade nature of these tumors [10,12,14,16,51-55].

The same surgical approach was used in Case 15 in our cohort, with no local recurrence or metastasis at 14 months follow-up. Zhang *et al*. reported a 33.3% contralateral recurrence rate after unilateral nephroureterectomy and demonstrated improved cancer-specific and overall survival with simultaneous bilateral nephroureterectomy without compromising graft function [16,20]. A particular malignancy evolution was observed in Case 3, where late donor-derived malignancy was suspected due to complete regression of pulmonary metastasis after allograft nephroureterectomy with bladder cuff for pT4HG UC and withdrawal of immunosuppression. Management of UTUC in graft kidneys is largely based on case reports and small retrospective series. Conservatory management (partial nephrectomy, ureteral resection) was also described in case reports [56-58].

This study has limitations, with potential selection bias due to its retrospective design and small sample size. Additionally, the lack of screening protocols for renal tumors in kidney transplant recipients may have led to the exclusion of some cases. Furthermore, the study did not identify the underlying cause of ESKD for all patients.

## CONCLUSION

In summary, RCC and UTUC in kidney transplant recipients present significant therapeutic challenges for the multidisciplinary team. Surgical management provides good oncologic outcomes with a low complication rate. It is important to highlight that screening for RCC and UC in kidney transplant recipients with associated risk factors may facilitate earlier diagnosis at lower stages and grades. Further prospective research or collaboration with larger multicentric cohorts could improve the elaboration of therapeutic and screening guidelines.

## References

[1] Karami S, Yanik EL, Moore LE, Pfeiffer RM, Copeland G, Gonsalves L (2016). Risk of renal cell carcinoma among kidney transplant recipients in the United States. Am J Transplant.

[2] Hickman LA, Sawinski D, Guzzo T, Locke JE (2018). Urologic malignancies in kidney transplantation. Am J Transplant.

[3] Butler AM, Olshan AF, Kshirsagar AV, Edwards JK, Nielsen ME, Wheeler SB, Brookhart MA (2015). Cancer incidence among US Medicare ESRD patients receiving hemodialysis, 1996-2009. Am J Kidney Dis.

[4] Au E, Wong G, Chapman JR (2018). Cancer in kidney transplant recipients. Nat Rev Nephrol.

[5] Sapir-Pichhadze R, Laprise C, Beauchamp ME, Kaouache M, Zhang X, Della Vecchia A, t al (2024). Immunosuppression and cancer risk in kidney transplant recipients: A retrospective cohort study. Int J Cancer.

[6] Al-Adra DP, Hammel L, Roberts J, Woodle ES, Levine D, Mandelbrot D (2021). Pretransplant solid organ malignancy and organ transplant candidacy: A consensus expert opinion statement. Am J Transplant.

[7] Dahle DO, Skauby M, Langberg CW, Brabrand K, Wessel N, Midtvedt K (2022). Renal Cell Carcinoma and Kidney Transplantation: A Narrative Review. Transplantation.

[8] Grulich AE, van Leeuwen MT, Falster MO, Vajdic CM (2007). Incidence of cancers in people with HIV/AIDS compared with immunosuppressed transplant recipients: A meta-analysis. Lancet.

[9] Engels EA (2019). Epidemiologic perspectives on immunosuppressed populations and the immunosurveillance and immunocontainment of cancer. Am J Transplant.

[10] Nortier JL, Martinez MC, Schmeiser HH, Arlt VM, Bieler CA, Petein M, Depierreux MF, De Pauw L, Abramowicz D, Vereerstraeten P, Vanherweghem JL (2000). Urothelial carcinoma associated with the use of a Chinese herb (Aristolochia fangchi). N Engl J Med.

[11] Zhang B, Shen C, Han WK, Yu W (2014). Comparison of clinicopathologic characteristics of urothelial carcinoma between patients after renal transplantation and on dialysis. Transplantation.

[12] Li XB, Xing NZ, Wang Y, Hu XP, Yin H, Zhang XD (2008). Transitional cell carcinoma in renal transplant recipients: a single center experience. Int J Urol.

[13] Green DA, Rink M, Xylinas E, Matin SF, Stenzl A, Roupret M (2013). Urothelial carcinoma of the bladder and the upper tract: disparate twins. J Urol.

[14] Rogers A, Ng JK, Glendinning J, Rix D (2012). The management of transitional cell carcinoma (TCC) in a European regional renal transplant population. BJU Int.

[15] Luo HL, Chiang PH, Cheng YT, Chen YT (2019). Propensity-Matched Survival Analysis of Upper Urinary Tract Urothelial Carcinomas between End-Stage Renal Disease with and without Kidney Transplantation. Biomed Res Int.

[16] Zhang Q, Ma R, Li Y, Lu M, Zhang H, Qiu M (2021). Bilateral Nephroureterectomy Versus Unilateral Nephroureterectomy for Treating De Novo Upper Tract Urothelial Carcinoma After Renal Transplantation: A Comparison of Surgical and Oncological outcomes. Clin Med Insights Oncol.

[17] Tai HC, Lai MK, Chung SD, Huang KH, Chueh SC, Yu HJ (2009). Intermediate-term oncological outcomes of hand-assisted laparoscopic versus open bilateral nephroureterectomy for dialysis and kidney transplant patients with upper urinary tract urothelial carcinoma. J Endourol.

[18] Chiang YJ, Yang PS, Wang HH, Lin KJ, Liu KL, Chu SH (2012). Urothelial cancer after renal transplantation: an update. Transplant Proc.

[19] Kojima Y, Takahi Y, Ichimaru N, Okumi M, Takahara S, Nonomura N (2015). Successful treatment of metastatic urothelial carcinoma arising in a transplanted renal allograft with paclitaxel, cisplatin, and gemcitabine combination therapy: a case report. BMC Res Notes.

[20] Du C, Zheng M, Wang Z, Zhang J, Lin J, Zhang L (2023). Clinical characteristics and treatment outcomes of kidney transplant recipients with de novo urothelial carcinoma: thirty years of experience from a single center. BMC Urol.

[21] Klatte T, Marberger M (2011). Renal cell carcinoma of native kidneys in renal transplant patients. Curr Opin Urol.

[22] Barama A, St-Louis G, Nicolet V, Hadjeres R, Daloze P (2005). Renal cell carcinoma in kidney allografts: a case series from a single center. Am J Transplant.

[23] Griffith JJ, Amin KA, Waingankar N, Lerner SM, Delaney V, Ames SA (2017). Solid Renal Masses in Transplanted Allograft Kidneys: A Closer Look at the Epidemiology and Management. Am J Transplant.

[24] Tillou X, Guleryuz K, Doerfler A, Bensadoun H, Chambade D, Codas R (2014). members of the Renal Transplantation Committee of the French Urological Association (CTAFU). Nephron sparing surgery for De Novo kidney graft tumor: results from a multicenter national study. Am J Transplant.

[25] Chambade D, Meria P, Tariel E, Vérine J, De Kerviler E, Peraldi MN (2008). Nephron sparing surgery is a feasible and efficient treatment of T1a renal cell carcinoma in kidney transplant: a prospective series from a single center. J Urol.

[26] Butler AM, Olshan AF, Kshirsagar AV, Edwards JK, Nielsen ME, Wheeler SB (2015). Cancer incidence among US Medicare ESRD patients receiving hemodialysis, 1996-2009. Am J Kidney Dis.

[27] Doublet JD, Peraldi MN, Gattegno B, Thibault P, Sraer JD (1997). Renal cell carcinoma of native kidneys: prospective study of 129 renal transplant patients. J Urol.

[28] Cheung CY, Lam MF, Lee KC, Chan GS, Chan KW, Chau KF (2011). Renal cell carcinoma of native kidney in Chinese renal transplant recipients: a report of 12 cases and a review of the literature. Int Urol Nephrol.

[29] Borga AL, Lima AC, Alves JCR, Deboni LM, Garcia CE, Guterres JCP (2022). Renal cell carcinoma in transplanted kidney: A case report and literature review. Brazil J Transplant.

[30] Webster AC, Craig JC, Simpson JM, Jones MP, Chapman JR (2007). Identifying high risk groups and quantifying absolute risk of cancer after kidney transplantation: a cohort study of 15,183 recipients. Am J Transplant.

[31] Wong G, Hayen A, Chapman JR, Webster AC, Wang JJ, Mitchell P, Craig JC (2009). Association of CKD and cancer risk in older people. J Am Soc Nephrol.

[32] Liang JA, Sun LM, Yeh JJ, Sung FC, Chang SN, Kao CH (2011). The association between malignancy and end-stage renal disease in Taiwan. Jpn J Clin Oncol.

[33] Schwarz A, Vatandaslar S, Merkel S, Haller H (2007). Renal cell carcinoma in transplant recipients with acquired cystic kidney disease. Clin J Am Soc Nephrol.

[34] Yanik EL, Clarke CA, Snyder JJ, Pfeiffer RM, Engels EA (2016). Variation in Cancer Incidence among Patients with ESRD during Kidney Function and Nonfunction Intervals. J Am Soc Nephrol.

[35] Lee HH, Choi KH, Yang SC, Han WK (2012). Renal cell carcinoma in kidney transplant recipients and dialysis patients. Korean J Urol.

[36] Al-Adra D, Al-Qaoud T, Fowler K, Wong G (2022). De Novo Malignancies after Kidney Transplantation. Clin J Am Soc Nephrol.

[37] Gigante M, Neuzillet Y, Patard JJ, Tillou X, Thuret R, Branchereau J (2012). members of the Comité de Cancerologie de l'Association Française d'Urologie (CCAFU); Comité de Transplantation de l'Association Française d'Urologie (CTAFU). Renal cell carcinoma (RCC) arising in native kidneys of dialyzed and transplant patients: are they different entities? BJU Int.

[38] Master VA, Meng MV, Grossfeld GD, Koppie TM, Hirose R, Carroll PR (2004). Treatment and outcome of invasive bladder cancer in patients after renal transplantation. J Urol.

[39] Cuenca AG, Rosales I, Lee RJ, Wu CL, Colvin R, Feldman AS (2020). Resolution of a High Grade and Metastatic BK Polyomavirus-Associated Urothelial Cell Carcinoma Following Radical Allograft Nephroureterectomy and Immune Checkpoint Treatment: A Case Report. Transplant Proc.

[40] Hirsch HH, Brennan DC, Drachenberg CB, Ginevri F, Gordon J, Limaye AP (2005). Polyomavirus-associated nephropathy in renal transplantation: interdisciplinary analyses and recommendations. Transplantation.

[41] Lamarche C, Orio J, Collette S, Senécal L, Hébert MJ, Renoult É (2016). BK Polyomavirus and the Transplanted Kidney: Immunopathology and Therapeutic Approaches. Transplantation.

[42] Chadban SJ, Ahn C, Axelrod DA, Foster BJ, Kasiske BL, Kher V (2020). KDIGO Clinical Practice Guideline on the Evaluation and Management of Candidates for Kidney Transplantation. Transplantation.

[43] Kalapara AA, Frydenberg M (2020). The role of open radical nephrectomy in contemporary management of renal cell carcinoma. Transl Androl Urol.

[44] Klatte T, Berni A, Serni S, Campi R (2021). Intermediate-and long-term oncological outcomes of active surveillance for localized renal masses: a systematic review and quantitative analysis. BJU Int.

[45] Nabavizadeh R, Noorali AA, Makhani SS, Hong G, Holzman S, Patil DH (2020). Transplant Radical Nephrectomy and Transplant Radical Nephroureterectomy for Renal Cancer: Postoperative and Survival Outcomes. Ann Transplant.

[46] DeLong MJ, Schmitt D, Scott KM, Ramakumar S, Lien YH (2003). Multicentric papillary renal carcinoma in renal allograft. Am J Kidney Dis.

[47] Andras I, Pecoraro A, Telecan T, Piana A, Boissier R, Hevia V (2023). en nombre del Grupo de Trasplante Renal y Cáncer Renal de la sección de Jóvenes Urólogos Académicos (YAU). How to manage renal masses in kidney transplant recipients? A collaborative review by the EAU-YAU kidney transplantation and renal cancer working groups. Actas Urol Esp (Engl Ed).

[48] Llamas F, Gallego E, Salinas A, Virseda J, Pérez J, Ortega A (2009). Sarcomatoid renal cell carcinoma in a renal transplant recipient. Transplant Proc.

[49] Tillou X, Doerfler A, Collon S, Kleinclauss F, Patard JJ, Badet L (2012). “Comité de Transplantation de l’Association Française d’Urologie (CTAFU)” De novo kidney graft tumors: results from a multicentric retrospective national study. Am J Transplant.

[50] Chambade D, Meria P, Tariel E, Vérine J, De Kerviler E, Peraldi MN (2008). Nephron sparing surgery is a feasible and efficient treatment of T1a renal cell carcinoma in kidney transplant: a prospective series from a single center. J Urol.

[51] Vaudreuil L, Bessede T, Boissier R, Bouye S, Branchereau J, Caillet K (2020). De novo renal carcinoma arising in non-functional kidney graft: a national retrospective study. Int Urol Nephrol.

[52] Tsaur I, Karalis A, Blaheta R, Juengel E, Vallo S, Scheuermann EH (2011). Transitional cell carcinoma of the native urinary tract after kidney transplantation: recommendations following a long-term retrospective analysis. Am J Med Sci.

[53] Thon WF, Kliem V, Truss MC, Anton P, Kuczyk M, Stief CG (1995). Denovo urothelial carcinoma of the upper and lower urinary tract in kidney--transplant patients with end-stage analgesic nephropathy. World J Urol.

[54] Hou HJ, Xiao J, Tian Y (2013). Contralateral nephroureterectomy for renal transplant recipients with unilateral upper urinary tract transitional cell carcinoma: a report of 12 cases. Transplant Proc.

[55] Ho CJ, Huang YH, Hsieh TY, Yang MH, Wang SC, Chen WJ (2023). New Hydronephrosis in the Native Kidney Is Associated with the Development of De Novo Urinary Bladder Urothelial Carcinoma in Patients with Post-Kidney Transplantation. Healthcare (Basel).

[56] Olsburgh J, Zakri RH, Horsfield C, Collins R, Fairweather J, O'Donnell P (2016). TCC in transplant ureter—When and when not to preserve the transplant kidney. Am J Transplant.

[57] Chiang YJ, Chu SH, Liu KL, Lai WJ, Wang HH (2006). Silent urothelial cancer detected by sonography after renal transplantation. Transplant Proc.

[58] Leon G, Szabla N, Boissier R, Gigante M, Caillet K, Verhoest G (2020). members of “Comité de Transplantation de l'Association Française d'Urologie” (CTAFU) Kidney Graft Urothelial Carcinoma: Results From a Multicentric Retrospective National Study. Urology.

